# New Structural State of Tungsten in Obtaining Porous Tungsten Coatings

**DOI:** 10.3390/ma17225591

**Published:** 2024-11-15

**Authors:** Yuriy Zhianshahovich Tuleushev, Valeriy Nicolaevich Volodin, Eldar Askhatovich Zhakanbaev, Bakytzhan Muratuly Tynyshbay

**Affiliations:** Institute of Nuclear Physics, Almaty 050032, Kazakhstan; yuriy.tuleushev@inp.kz (Y.Z.T.); volodin@inp.kz (V.N.V.); zhakanbayev.yeldar@inp.kz (E.A.Z.)

**Keywords:** tungsten, lead, magnetron sputtering, X-ray structural studies

## Abstract

For the first time, porous tungsten coatings were obtained by vacuum annealing of coatings consisting of both lead solution in β-W and a mixture of Pb solution in β-W and pure lead. Electron microscopic studies of the obtained coatings before and after vacuum annealing at 800 °C for 1 h and 6 h were carried out. The formation of a new phase of tungsten with an octa-lattice and parameter a = 0.4878 ± 0.0005 nm was recorded.

## 1. Introduction

Our interest in the study of tungsten–lead alloys is due to the fact that composite alloys based on tungsten, as well as tungsten oxides, are widely used for radiation applications in such industries as medicine, agriculture, energy, and industry [1,2,3,4,5,6]. The authors of [7] reported a newly developed lead–tungsten–boron nuclear shielding glass system for use in radiation applications against photons, neutrons, and charged particles. The Bi-doped lead–tungsten–boron composite system exhibits promising performance characteristics better than many commercial and conventional systems and heavy concretes. Of all metal oxides, tungsten (WO) oxide (WO_3_) is considered a highly significant semiconductor material with promising potential for photoelectrochemical water splitting [8]. The creation of porous composite materials is also a topical task for the creation of new classes of sensor materials. For example, the porous tungsten emitter is a core component of field emission electric propulsion [9]. A porous tungsten–epoxy composite has been established that is used in many applications, including X-ray and γ-ray shielding, for example, in concrete monitoring sensors [10,11,12]. The authors of [13] highlighted the technology of the synthesis of porous tungsten by low-temperature sintering for cathodes in dispensers, which is used in electronic valves and high-power lamps.

However, information regarding pure tungsten–lead alloy with a high lead content is very limited [14]. When conducting research on the magnetron synthesis of a porous coating based on W-Pb, similar to what we carried out in [15], we discovered a new modification of the tungsten crystal lattice. Initially, tungsten–lead coatings with different lead contents from 26.2 at.% to 49.5 at.% were synthesized by using a magnetron-sputtering deposition system. A study of the structural and phase composition of these coatings showed that they mainly involve a solid solution of lead in tungsten and pure lead. Next, in order to form a porous structure, it was necessary to remove lead from the coating by thermal vacuum annealing. When removing lead from a solution of tungsten in lead by vacuum annealing at T = 800 °C, we detected an anomaly in the behavior of the tungsten crystal lattice. Tungsten atoms remained in the places in which they were, as they were in a solid solution of tungsten in lead, and retained the crystal lattice parameters of the solid solution of lead in tungsten, but no lead should remain in the sample in accordance with the heat treatment conditions. In addition, tungsten, during heat treatment, had to change its structural state, returning to the parameters of the crystalline cell of pure tungsten. In previous studies [16], we carried out work on the production of coatings from porous tungsten by the vacuum annealing of coatings of the W-Cd system, but no new modifications of the lattice of pure tungsten were observed since cadmium has a hexagonal lattice and does not dissolve tungsten in itself at all. For a more detailed study of this effect, we carried out X-ray structural studies with complementary studies using a scanning electron microscope.

## 2. Experimental Details

A magnetron-sputtering system with two independent DC magnetron devices was used to prepare samples of tungsten–lead system coatings. The vacuum chamber was evacuated to a pressure of 3 × 10^−3^ Pa, and then argon was injected into the chamber to a pressure of 1 × 10^−1^ Pa, which is the working pressure for ignition of the magnetron device. The working gas used for magnetron sputtering was argon. In the experiments, we used the magnetrons equipped with two targets: tungsten 99.96 mas. % and lead (99.99 wt.%). The study used a power supply for magnetrons of its own, made using a transformerless circuit of a thyristor pulse-width power regulator, connected to an alternating current network with a voltage of 220 V [17]. The range of powers in a magnetron device was from 80 to 100 watts for tungsten and from 7.4 to 15.3 watts for lead. We used polycor (α-Al_2_O_3_) as substrates on which coatings were deposited. A cylinder mounted inside the vacuum chamber with the ability to rotate around an axis to secure the substrates and move them relative to the magnetrons was used. A rotation speed of 10 revolutions per minute was selected to prepare samples of coatings of the tungsten–lead system. Thus, during the sputtering time, which for coatings of this system was 40–60 min, each substrate passed by the tungsten and lead magnetrons sputtering 400–600 times, so the coating was formed layer by layer. This approach to coating formation allows metals to be mixed in the coating in almost any concentration. The experimental device is described in more detail in [15,16]. The ratio and thickness of the deposited components were controlled by weight—by the amount of sputtered and deposited metal during the formation of the coating and, in parallel, by the Rutherford backscattering of N^14^ ions on a DC-60 accelerator from the Eurasian University (Nur-Sultan, Kazakhstan). X-ray diffraction (XRD) measurements were conducted on a Bruker D8 “Discover” X-ray diffractometer. The data were collected as Bragg–Brentano geometry (Θ–2Θ) over an angular range of 2Θ = 20–90° with a step size of 0.01°. Electron microscopy studies used a JSM-8230 electron probe microanalyzer (JEOL, Japan). High-temperature annealing was performed in a vacuum furnace, designed based on the URVT-2500 system. The annealing temperature was selected experimentally, and the obtained tungsten–lead coatings were annealed from 300 °C and with a step of 50 °C, up to 800 °C. The achievement of the required temperature was determined by the results of X-ray structural studies, based on the disappearance of lead reflections in diffraction patterns.

## 3. Results and Discussion

**Figure 1:**
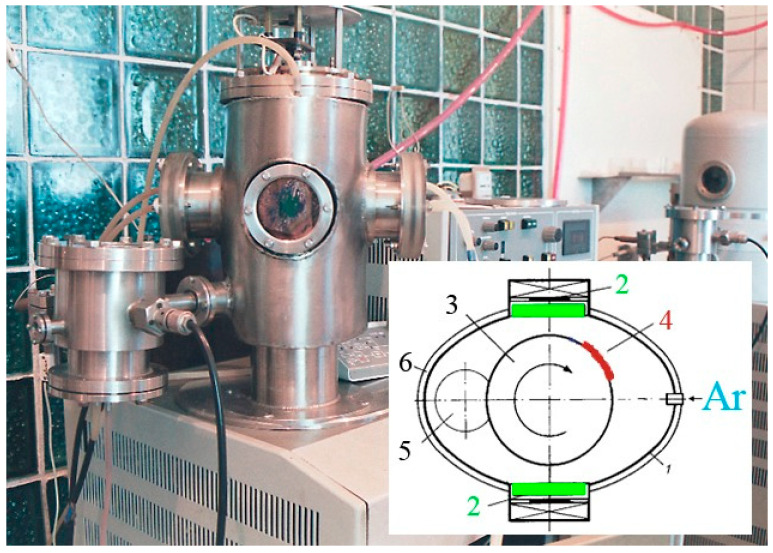
Setup for forming coatings of the tungsten-lead system: 1—vacuum chamber body; 2—magnetrons; 3—cylinder; 4—substrate; 5—gas evacuation window; 6—device body.

Using the setup shown in Figure 1, coatings were sputtered onto the polycor (α-Al_2_O_3_) substrates of three different compositions, details of which are presented in Table 1.

The obtained coatings were annealed in an oil-free vacuum at 800 °C for 1 h and 6 h, after which the annealed and non-annealed samples were diffractometrically investigated on a Bruker D8 Advance diffractometer, the results of which are given below.

**Figure 2:**
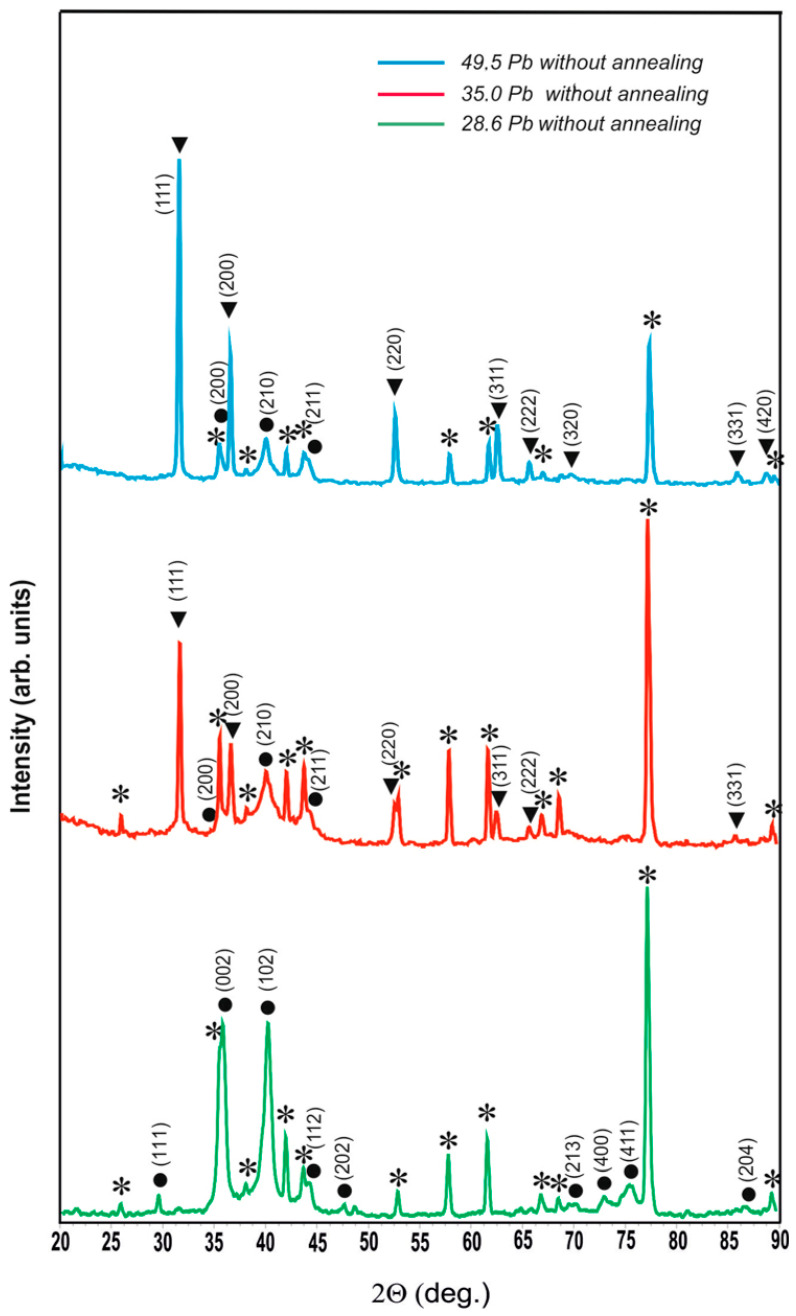
Diffractograms of non-annealed coatings of W-Pb system with different lead content: ⁎—polycor (α-Al_2_O_3_), ●—solid solution of lead in β-W, ▼—lead.

Figure 2 shows the diffractograms of unannealed coatings of the W-Pb system with a lead content of 28.6, 35.0, and 49.5 at.%. From the comparison of the diffractograms, it can be seen that at the concentration of 28.6 at.% lead is completely dissolved in β-W. The cubic lattice of β-W undergoes tetragonal distortion, and the calculated lattice parameters are a = 0.5220 nm and c = 0.5012 nm. When the lead content in the coating increases to 35.0 at.%, it begins to crystallize as a separate phase with tabulated lattice parameters. The phase of lead solution in β-W becomes finely crystalline and is well enough described in cubic syngony, with a lattice parameter of a = 0.5077 ± 0.0011 nm. With a further increase in lead concentration in the coating up to 49.5 at.%, the ratio between pure lead and the lead solution in β-W shifts even more strongly in favor of lead; the lead solution in β-W has the cubic lattice A15 with the parameter a = 0.5063 ± 0.0005 nm.

To test the possibility of obtaining porous structures from tungsten, the coatings of the W-Pb system both completely consisting of a solid solution of Pb in W (28.6 at.% Pb) and with an excess of lead as well as an excess of lead (35.0 and 49.5 at.% Pb) were annealed in vacuum at T = 800 °C. The annealing temperature was chosen to be obviously higher than the boiling point of lead in order to achieve the complete evaporation of lead during annealing.

**Figure 3:**
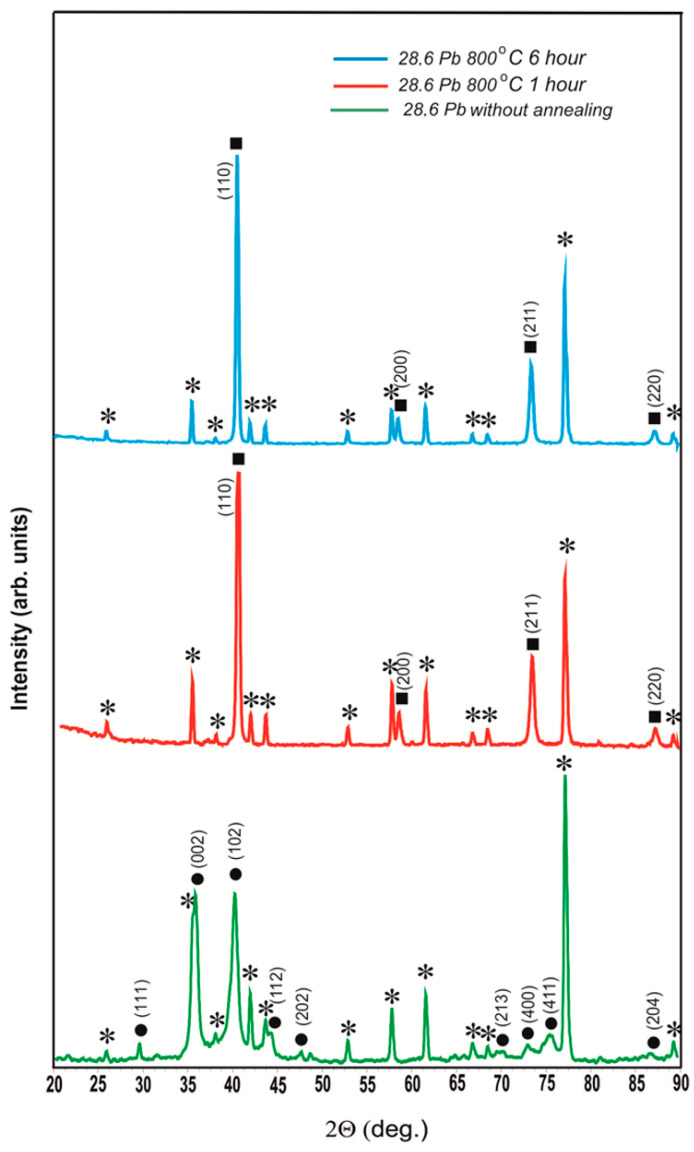
Diffractograms of the coating with 28.6 at.% lead content without annealing and after annealing at 800 °C for 1 h and 6 h: ⁎—polycor (α-Al_2_O_3_); ●—a solid solution of Pb in β-W (a = 0.5220 nm, c = 0.5012 nm); ■—α-W (a = 0.3163 ± 0.0002 nm).

Figure 3 shows the diffractograms of the coating with a lead content of 28.6 at.% without annealing and after annealing at 800 °C for 1 h and 6 h.

As can be seen from Figure 3, as a result of annealing 800 °C 1 h, the solid solution of lead in β-W completely transformed into α-W with a lattice parameter of a = 0.3163 ± 0.0002 nm. After annealing at 800 °C for 6 h, the X-ray diffraction pattern completely coincides with the X-ray diffraction pattern after annealing at 800 °C for 1 h. Therefore, according to the data of X-ray radiography, lead is completely evaporated from the coating with a thickness of 2.6 μm in vacuum at T = 800 °C for 1 h.

The analysis of the diffractograms after annealing at 800 °C for 1 h and 6 h shows that, in addition to the peaks from α-W with the tabulated lattice parameter and polycor (α-Al_2_O_3_), there is a system of peaks with *d_hkl_*, as presented in Table 2.

It can be seen that if the Miller indices in the second row of the table are assigned to the observed *d_hkl_*, it turns out that we observe an OCC lattice with the parameter a = 0.4878 ± 0.0005 nm. The coincidence of diffractograms after annealing at 800 °C for 1 h and 6 h shows that the obtained phase is stable in time.

**Figure 4:**
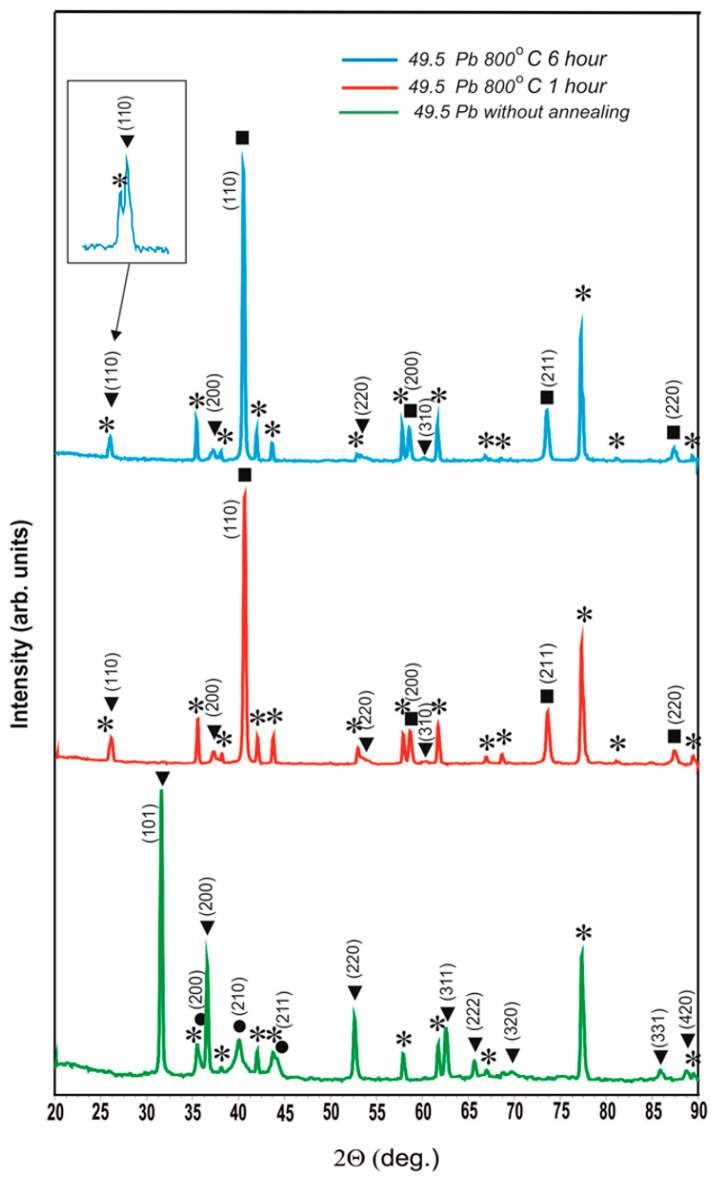
Diffractograms of the coating with a lead content of 49.5 at.% without annealing and after annealing at 800 °C for 1 h and 6 h: ⁎—polycor (α-Al_2_O_3_); ●—solid solution of Pb in β-W (a = 0.5077 ± 0.0011 nm); ■—α-W (a = 0.3163 ± 0.0002 nm); ▼—OCC phase (a = 0.4878 ± 0.0005 nm).

Figure 4 shows the diffractograms of the unannealed coating containing 49.5 at.% Pb and the same coating after annealing at 800 °C for 1 h and 6 h.

**Figure 5:**
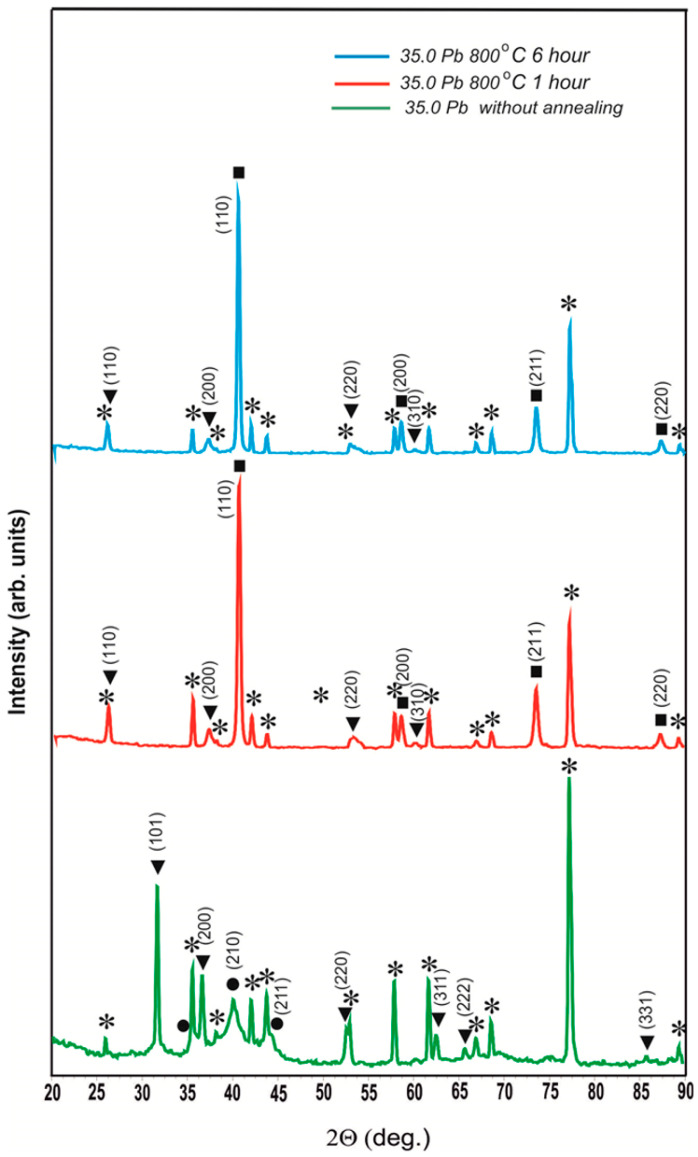
Diffractograms of the coating with 35.0 at.% lead content without annealing and after annealing at 800 °C for 1 h and 6 h: ⁎—polycor (α-Al_2_O_3_); ●—solid solution of Pb in β-W (a = 0.5077 ± 0.0011 nm); ■—α-W (a = 0.3163 ± 0.0002 nm); ▼—OCC phase (a = 0.4876 ± 0.0009 nm).

The diffractograms of this coating after 800 °C annealing for 1 h and 6 h completely coincide with the diffractograms of the coating with a Pb content of 35.0 at.%. Therefore, it can be concluded that the existence of the OCC phase with a = 0.4878 ± 0.0005 nm after 800 °C annealing is a general pattern for coatings in which lead existed as a separate phase before annealing. In Figure 5, we particularly used an enlarged scale so that no one doubts the reality of the existence of the peak (110) of this phase. 

Moreover, it is necessary to note the fact that the lattice parameter of the detected phase a = 0.4878 ± 0.0005 nm is only slightly less than the lattice parameter of lead (a_Pb_ = 0.4950 nm). The reasons for the occurrence of this phase, in our opinion, are as follows: during the sputtering of coatings of the tungsten–lead system at a lead concentration of less than 28.6 at.%, a solid solution of lead in β-W is generated. Above this threshold, some of the lead crystal transitions into a separate phase. A minor part of this lead dissolves in tungsten because of nanoscale alloying and forms a solid solution of tungsten in lead. On the diffractograms of Figure 1, the existence of a solid solution of tungsten in lead cannot be confirmed due to the fact that a weak reflex from a solid solution of tungsten in lead exists in the form of a weak distortion on the right part of peaks from pure lead. When heated at 800 °C in vacuum, all of the lead—free, dissolved in β-W, and dissolved in tungsten—is vaporized. Where there was free lead, cavities remained. Lead from β-W evaporated with a decrease in the volume of the latter, and cracks were formed in β-W as in clay when it dries in the sun. Regarding the evaporation of the lead dissolved in tungsten, tungsten atoms remained in the positions of the lattice where they were in solid solution, and therefore a = 0.4878 ± 0.0005 nm. However, in the absence of a force field of lead atoms, the characteristic of the interaction of tungsten atoms among themselves changes, and on the diffractogram, we observe the OCC lattice. Thus, these results confirm tungsten dissolution in lead and the ability of atoms of the dissolved substance to memorize their position in the solvent lattice when the solvent atoms are removed from it.

**Figure 6:**
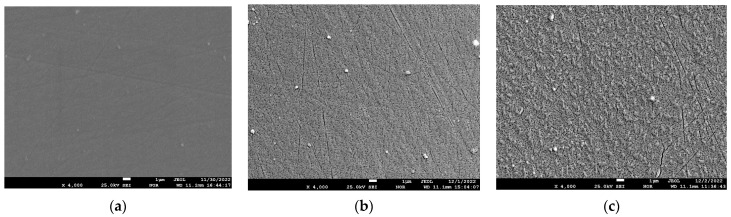
Electron microscopic images of the surface of the sample with a lead content of 28.6 at.% before annealing (**a**) and after annealing at 800 °C for 1 h (**b**) and 6 h (**c**).

To verify the above assumptions, electron microscopic studies were carried out on an electron probe microanalyzer JSM-8230 (JEOL). Figure 6 shows the electron microscopic images of the surface of the sample with a lead content of 28.6 at.% before annealing and after annealing at 800 °C for 1 h and 6 h.

The analysis of Figure 6 shows that, before annealing, the surface of the coating is flat without any features, and even stripes can be seen on the left side of the surface of the polycor after polishing. After annealing at 800 °C for 1 h (Figure 6b), many small pores and cracks on the coating surface can be seen. After annealing at 800 °C for 6 h (Figure 6c), it can be seen that surface roughness due to a reduction in the coating volume increased, and the number of pores and the size of the cracks covering the surface also increased.

Table 3 shows the results of the EDS mapping of the sample with a lead content of 28.6 at.% after annealing at 800 °C for 6 h (c).

From the EDS mapping results, it can be seen that lead completely evaporates from the coating after annealing at 800 °C for 6 h. Obviously, the elements Al, O, and C are related to the substrate (Al_2_O_3_ polycor), and tungsten is completely free of lead.

**Figure 7:**
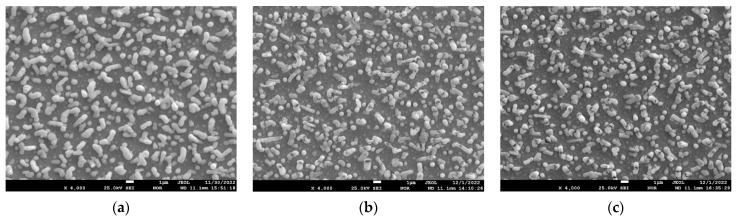
Electron microscopic images of the surface of the sample with a lead content of 35 at.% before annealing (**a**) and after annealing at 800 °C for 1 h (**b**) and 6 h (**c**).

Figure 7 shows the electron microscopic images of the surface of the sample with a lead content of 35.0 at.% before annealing and after annealing at 800 °C for 1 h and 6 h.

**Figure 8:**
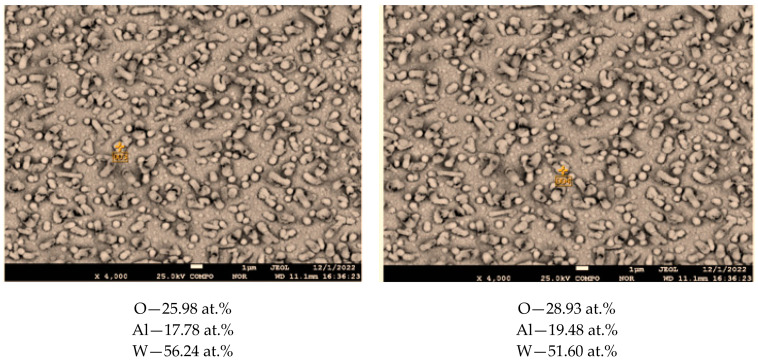
EDS analysis data at different points of the coating surface with 35 at.% Pb content.

From the analysis of Figure 7, it can be seen that the coating surface after sputtering and before annealing is dense and smooth, and numerous lead whiskers long enough stick out of it. After annealing at 800 °C 1 h (Figure 7b), it can be seen that there are many small pores and cracks in the coating surface, holes are formed in the whisker walls, and the whiskers are empty inside. After annealing at 800 °C for 6 h (Figure 6c), it can be seen that the porosity of the coating between the whiskers increases, while the whisker shells remain unchanged. To understand the nature of the whiskers remaining after annealing at 800 °C for 6 h, let us consider the data from the spot EDS analysis at different points on the surface. Figure 8 shows the images of the 35.0 at.% coating surface with the elemental composition at these points.

Aluminum and oxygen refer to the substrate consisting of α-Al_2_O_3_. Since even when pointing directly at the surface of the whisker, the instrument did not detect the presence of lead, it can be concluded that the outer surface of the whisker was formed by a solid solution of tungsten in lead, and it was the tungsten crust that remained after lead evaporated. From the analysis of this image, it can be seen that the average diameter of the lead whiskers is approximately 500–600 nm, and the wall thickness of these whiskers, which can be estimated from the analysis of the exposed whiskers, is 20–30 nm. To estimate the size of crystallites forming the walls of the phase whiskers, we used the Scherrer formula $d=\frac{K\;\lambda }{\cos\Theta }$, where d is the average size of crystallites, K is the dimensionless particle shape factor (Scherrer constant), λ is the wavelength of X-ray radiation, β is the width of the reflex at half-height (in radians), and Θ is the diffraction angle (Bragg angle). The program Eva-2 from the Bruker D8 Advance diffractometer software package was used to determine the peak (200) of the X-ray diagram of the sample with 49.5 at.% Pb after annealing at 800 °C for 6 h (Figure 5, upper spectrum), which revealed a crystallite size equal to 24.9 nm.

**Figure 9:**
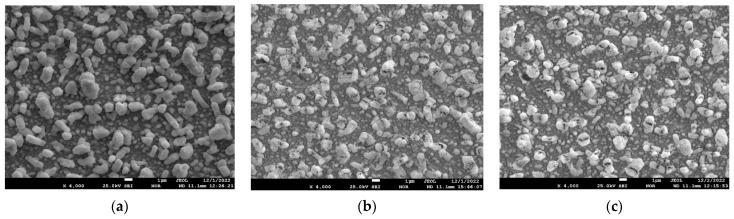
Electron microscopic images of the surface of the sample with lead content 49.5 at.% before annealing (**a**) and after annealing at 800 °C for 1 h (**b**) and 6 h (**c**).

Figure 9 shows the electron microscopic images of the surface of the sample with a lead content of 49.5 at.% before annealing and after annealing at 800 °C for 1 h and 6 h.

From the analysis of Figure 9, it can be seen that the number of lead whiskers per unit surface increases, and the whiskers themselves become thicker. After annealing at 800 °C for 1 h (Figure 7b), it can be seen that there are many small pores on the coating surface, holes are formed in the whisker walls, and the whiskers are empty inside. After annealing at 800 °C for 6 h (Figure 6c), it can be seen that the porosity of the coating between the whiskers increases, while the whisker shells remain unchanged. It is quite obvious that the porosity of the coating with an initial lead concentration of 49.5 at.% after annealing 800 °C 6 h is maximum.

Thus, the possibility of obtaining porous tungsten coatings by vacuum annealing of W-Pb system coatings at 800 °C with lead contents of 28.6, 35.0, and 49.5 at.% was confirmed. In this case, the nature of the porosity changes, and if the coating with 28.6 at.% is characterized by deep cracks on the surface and multiple pinpoint pores, for the coating with 49.5 at.%, the surface becomes spongy.

In addition, the formation of a new tungsten phase during lead evaporation from a solid solution of tungsten in lead forming on the outer walls of lead whiskers growing on the surface of sputtered coatings was identified.

## 4. Conclusions

Studies were carried out on obtaining porous tungsten coatings by vacuum annealing of W-Pb coatings with different lead content. It was found that at a lead concentration of up to 28.6 at.%, lead dissolved in β-W, and at a lead concentration of 35.0 and 49.5 at.% in the coating, a part of lead was isolated in the form of a separate phase, which appeared as rather long whiskers of rounded shape. On annealing in vacuum at T = 800 °C, the lead from the 28.6 at.% Pb coating evaporated and converted to pure α-W. The tungsten surface exhibited deep cracks, and between them, there were many pinpoint pores. When the 35.0 and 48.5 at.5 at.% Pb coatings were annealed in vacuum at T = 800 °C, and the β-W solution of lead in them converted into α-W, the lead contained in the whiskers evaporated, and the whisker walls were preserved but in the form of very friable formations consisting of tungsten in a new crystallographic modification with an OCC lattice with a parameter of a = 0.4878 ± 0.0005 nm. A comparison of annealing in vacuum at T = 800 °C for 1 h and 6 h shows that the obtained tungsten modification was stable over time.

Previously, the authors of this article conducted work on obtaining coatings from porous tungsten during vacuum annealing of coatings of the W-Cd system, but new modifications of tungsten did not occur, since cadmium has a hexagonal lattice and it does not dissolve tungsten in itself at all.

This work was supported by the MES RK program BR18574073.

## Figures and Tables

**Table 1:** The P_W_ and P_Pb_ powers used in tungsten and lead sputtering, the recorded molar removal from each target n_W_ and nPb, the thicknesses of the tungsten and lead partial layers d_W_ d_Pb_, the total coating thickness d, and the calculated lead concentration in the C_Pb_ coating.

P_W_, Watt	P_Pb_, Watt	N_W_, mmol	n_Pb_, mmol	d_W_, nm/layer	d_Pb_, nm/layer	Thickness d, µm	C_Pb_, at.%	Structure
100	7.4	4.528	1.818	2.4	1.9	2.55	28.6	solid solution W-Pb a = 5.048 ± 0.013 A
100	10	4.040	2.179	2.0	2.2	2.61	35.0	Pb + solid solution W-Pb a = 5.077 ± 0.011 A
80	15.3	5.589	3.517	1.90	3.57	3.28	49.5	Pb + solid solution W-Pb a = 5.067 ± 0.004 A

**Table 2:** The observed *d_hkl_* of the unknown phase and inferred Miller indices *(hkl)*.

*d_hkl_*, nm	0.3447	0.2434	0.1728	0.1545
*(hkl)*	(110)	(200)	(220)	(310)

**Table 3:** EDS mapping results of the sample with a lead content of 28.6 at.% after annealing at 800 °C for 6 h (c).

Element	Mass (%)	Atom (%)	K (%)
C	5.195	25.4630	0.8454
O	10.262	37.7554	1.922
Al	5.219	11.3850	2.612
W	79.324	25.3965	60.502

## Data Availability

The original contributions presented in the study are included in the article, further inquiries can be directed to the corresponding author.
